# Supplementary material for: A Likelihood Approach for Real-Time Calibration of Stochastic Compartmental Epidemic Models
Source: PLoS Comput Biol. 2017 Jan 17;13(1):e1005257. doi: 10.1371/journal.pcbi.1005257 (PMC5240920; doi:10.1371/journal.pcbi.1005257)
Supplement: S1 File — (TAR.GZ) [file pcbi.1005257.s014.tar.gz › HSPH_Online-SI-Revision/EnKF07/n1-mild/n1-mild_EnKF_table-8.pdf]

1	R0	{1.27677, 7.89375}	{2.56117, 7.27119}	{{1.65069, 2.5413}, {3., 12.7711}}	{100.597, 46.0358}	inf	9.	190.215	{61.1163, 325.468}	2017.98	1.18787	newly	11.	180.32	{39., 363.}	1545.82	1.06942	Reff	1.2423	0.672052	{0.288094, 0.993585}	57.2214	ar	400.	1563.15	{1193., 1965.}	290.788
2	R0	{1.33231, 10.5259}	{2.47553, 7.04685}	{{1.63011, 3.41038}, {3., 13.3802}}	{90.556, 41.7041}	inf	43.	54.6124	{10.4866, 73.0031}	89.7939	0.30381	newly	32.	62.45	{6., 58.}	153.344	0.361286	Reff	1.07517	0.459852	{0.0477496, 0.685442}	62.995	ar	467.	1818.1	{1645., 1948.}	289.315
3	R0	{1.2273, 6.76944}	{1.75038, 9.5006}	{{0.382072, 2.00079}, {3., 18.14}}	{78.507, 85.4191}	inf	19.	70.514	{14.6475, 151.011}	284.862	0.503486	newly	19.	147.15	{7., 412.}	695.737	0.652161	Reff	1.19048	1.40806	{0.317114, 2.57434}	64.3346	ar	314.	635.7	{155., 990.}	126.217
4	R0	{1.41215, 17.5742}	{2.25423, 6.87125}	{{1.22727, 2.61562}, {3., 14.5414}}	{71.3705, 62.1747}	inf	19.	136.601	{38.6217, 295.221}	620.38	0.725377	newly	6.	125.56	{26., 243.}	1993.67	1.1321	Reff	1.34578	0.457672	{0.122507, 1.09323}	68.2328	ar	465.	1575.59	{1143., 1954.}	238.837
5	R0	{1.42222, 6.15886}	{2.52638, 6.95132}	{{2.11525, 2.55486}, {3., 12.6205}}	{85.4068, 43.3413}	inf	13.	211.541	{61.9303, 376.546}	1529.21	1.10943	newly	24.	219.14	{60., 550.}	816.083	0.818415	Reff	1.34115	0.799607	{0.222402, 1.51066}	50.7781	ar	490.	1561.84	{1081., 2093.}	219.343
6	R0	{1.24363, 9.95596}	{1.77844, 9.04943}	{{0.416104, 2.00079}, {3., 17.6994}}	{78.7949, 48.5465}	inf	3.	75.5783	{14.8175, 151.91}	2425.09	1.2394	newly	3.	184.41	{10., 594.}	6056.33	1.39491	Reff	1.23741	1.43745	{0.329157, 2.68647}	62.5363	ar	411.	629.63	{136., 984.}	88.0365
7	R0	{1.36478, 5.96056}	{2.3267, 9.05791}	{{0.828735, 2.14249}, {3., 13.4356}}	{86.608, 77.8768}	inf	14.	198.557	{34.2609, 449.499}	1319.86	0.995936	newly	23.	228.85	{20., 628.}	903.957	0.811721	Reff	1.32384	1.38799	{0.213561, 2.39785}	53.7037	ar	484.	1131.18	{511., 2050.}	138.426
8	R0	{1.36335, 17.6429}	{2.41193, 6.76689}	{{1.61101, 4.09812}, {3., 9.29859}}	{78.1111, 61.6452}	inf	13.	59.9	{12.631, 139.623}	368.598	0.451984	newly	3.	68.3	{8., 119.}	2179.33	0.987832	Reff	1.33472	0.538817	{0.0789293, 1.32323}	64.7809	ar	484.	1872.81	{1652., 2323.}	287.267
9	R0	{1.26575, 5.28707}	{2.18442, 4.42849}	{{0.634452, 2.34239}, {3., 9.16651}}	{99.5287, 52.8274}	inf	7.	64.3282	{3.50142, 121.094}	835.202	0.641806	newly	8.	75.48	{4., 105.}	860.75	0.609232	Reff	1.13918	0.387685	{0.0881959, 0.64993}	70.9162	ar	472.	1855.89	{1665., 2385.}	293.197
10	R0	{1.42942, 9.49905}	{2.46908, 6.5528}	{{1.63561, 4.27727}, {3., 8.91008}}	{76.3522, 38.6448}	inf	25.	57.1422	{10.0153, 85.5461}	156.081	0.301489	newly	22.	64.12	{5., 96.}	233.727	0.387215	Reff	1.26932	0.437134	{0.0224507, 0.649308}	69.9529	ar	468.	1842.72	{1654., 2261.}	293.744
11	R0	{1.46827, 15.0983}	{2.29997, 4.71367}	{{0.625619, 2.08543}, {3., 10.3346}}	{83.0548, 68.7801}	inf	7.	39.2797	{4.72931, 50.228}	476.503	0.557965	newly	4.	46.14	{5., 48.}	1064.5	0.774882	Reff	1.43009	0.384621	{0.0930735, 0.681828}	74.703	ar	495.	1763.38	{1632., 1855.}	256.238
12	R0	{1.25751, 13.279}	{2.47274, 6.65848}	{{1.64956, 4.00573}, {3., 9.16616}}	{98.0717, 52.099}	inf	9.	71.3014	{9.32384, 260.553}	702.436	0.637573	newly	6.	85.55	{7., 263.}	1336.17	0.763046	Reff	1.20847	0.527746	{0.0349707, 1.2552}	65.2406	ar	366.	1898.76	{1656., 2476.}	418.787
13	R0	{1.3425, 9.16361}	{2.53203, 6.16053}	{{2.13031, 0.983777}, {3., 9.32475}}	{91.0382, 38.3996}	inf	36.	69.517	{14.2598, 176.261}	120.373	0.304205	newly	33.	109.7	{10., 295.}	270.242	0.413358	Reff	1.12233	0.689052	{0.114157, 2.12818}	64.6522	ar	442.	1794.13	{1586., 2504.}	306.64
14	R0	{1.18936, 6.20583}	{2.53736, 7.06583}	{{2.11072, 2.53158}, {3., 12.9625}}	{119.014, 44.8737}	inf	20.	227.426	{59.4594, 380.358}	1040.52	0.945411	newly	20.	230.51	{58., 583.}	1056.55	0.90827	Reff	1.1287	0.822984	{0.231823, 1.54185}	47.3203	ar	382.	1593.39	{1080., 2208.}	317.118
15	R0	{1.27137, 9.78868}	{2.52605, 6.96553}	{{2.11863, 2.56175}, {3., 12.6276}}	{106.266, 39.4976}	inf	14.	212.259	{63.4961, 381.306}	1417.44	1.07744	newly	12.	219.01	{59., 561.}	1726.25	1.107	Reff	1.20017	0.806698	{0.2323, 1.5255}	48.1676	ar	496.	1565.47	{1086., 2090.}	216.216
16	R0	{1.23102, 13.6452}	{2.22466, 7.31137}	{{1.20962, 4.28111}, {3., 14.7441}}	{89.7158, 53.3258}	inf	23.	138.747	{29.5554, 276.412}	511.505	0.655607	newly	11.	120.46	{15., 285.}	1000.18	0.84535	Reff	1.16085	0.51771	{0.135484, 1.1787}	63.7555	ar	445.	1598.54	{1144., 2041.}	259.222
17	R0	{1.27213, 13.7452}	{2.24176, 6.73204}	{{0.947645, 2.32019}, {3., 15.8104}}	{91.5913, 58.9267}	inf	8.	269.817	{64.6097, 511.829}	3272.71	1.42929	newly	5.	346.05	{54., 774.}	6821.	1.66393	Reff	1.2505	1.01829	{0.361276, 1.81617}	35.7694	ar	367.	1329.65	{704., 1884.}	262.401
18	R0	{1.25318, 11.2151}	{2.24666, 4.66321}	{{0.628821, 2.3392}, {3., 10.1103}}	{104.013, 59.3644}	inf	12.	63.4561	{4.04801, 116.39}	454.002	0.479938	newly	5.	71.5	{4., 68.}	1342.8	0.750315	Reff	1.17924	0.410826	{0.0988386, 0.642709}	69.8909	ar	416.	1842.66	{1655., 2214.}	342.947
19	R0	{1.46006, 8.11496}	{2.26822, 6.69192}	{{0.946958, 2.31902}, {3., 15.8146}}	{75.4771, 53.6051}	inf	12.	270.292	{64.569, 511.793}	2152.43	1.25296	newly	15.	345.92	{56., 795.}	2207.47	1.19132	Reff	1.43232	1.01133	{0.365154, 1.73269}	39.2099	ar	496.	1333.27	{702., 1871.}	169.603
20	R0	{1.14273, 6.71261}	{2.25551, 7.34579}	{{1.22179, 4.3972}, {3., 14.7243}}	{105.315, 36.6513}	inf	10.	129.336	{27.945, 247.239}	1195.72	0.949538	newly	11.	110.95	{20., 256.}	913.364	0.806076	Reff	1.10273	0.46439	{0.140284, 0.923451}	63.849	ar	362.	1563.05	{1196., 1821.}	331.782
21	R0	{1.42432, 8.23905}	{2.26131, 7.02914}	{{0.815504, 3.05843}, {3., 16.4293}}	{72.0662, 49.6478}	inf	12.	147.617	{12.0964, 378.463}	1141.07	0.934132	newly	16.	136.68	{9., 403.}	770.25	0.788576	Reff	1.21067	0.491031	{0.050995, 0.818462}	66.5235	ar	405.	1603.65	{1146., 2015.}	295.963
22	R0	{1.29386, 17.863}	{2.61573, 7.06545}	{{2.15802, 2.51007}, {3., 13.0337}}	{104.388, 60.4465}	inf	15.	214.522	{65.8731, 362.597}	1331.28	1.05898	newly	3.	229.51	{56., 551.}	7550.33	1.72223	Reff	1.26151	0.861538	{0.259573, 1.5884}	48.2201	ar	488.	1581.57	{1094., 2203.}	224.715
23	R0	{1.39274, 13.0167}	{2.47073, 6.99046}	{{1.64045, 3.6857}, {3., 10.9577}}	{79.7302, 47.49}	inf	20.	58.6629	{9.03329, 99.9929}	214.667	0.335213	newly	9.	64.1	{6., 120.}	625.111	0.533333	Reff	1.22283	0.462024	{0.0329477, 0.644387}	68.0669	ar	475.	1824.53	{1639., 2267.}	284.112
24	R0	{1.32178, 15.8991}	{2.02822, 4.32047}	{{0.213149, 1.54304}, {3., 5.96769}}	{96.1287, 72.8257}	inf	13.	123.568	{9.53719, 348.961}	861.736	0.769304	newly	5.	137.96	{10., 330.}	2662.	1.13553	Reff	1.27023	0.380734	{0.0383644, 0.617109}	70.8361	ar	471.	1640.45	{1016., 1994.}	248.49
25	R0	{1.37568, 17.8427}	{2.22765, 4.55024}	{{0.66478, 1.36564}, {3., 10.8796}}	{90.7369, 74.498}	inf	15.	63.6175	{5.46188, 63.6773}	349.657	0.457242	newly	9.	78.65	{4., 66.}	789.889	0.612564	Reff	1.2615	0.409418	{0.11295, 0.626938}	71.7912	ar	398.	1804.51	{1632., 2106.}	353.394
26	R0	{1.20993, 6.4656}	{1.96536, 4.98571}	{{0.220329, 1.69724}, {3., 7.51423}}	{106.218, 38.4475}	inf	16.	91.9205	{8.88176, 157.575}	489.311	0.651942	newly	14.	94.74	{10., 190.}	592.	0.693295	Reff	1.13007	0.362546	{0.0415902, 0.539867}	69.35	ar	345.	1655.12	{1514., 1908.}	379.768
27	R0	{1.20548, 13.8003}	{2.17571, 4.34014}	{{0.620764, 1.80043}, {3., 10.1654}}	{106.702, 68.5504}	inf	23.	51.624	{5.26932, 50.2253}	170.523	0.353452	newly	9.	63.41	{5., 59.}	619.	0.534206	Reff	1.15003	0.381166	{0.0948718, 0.674157}	71.8507	ar	350.	1808.6	{1660., 1905.}	416.743
28	R0	{1.3176, 14.6594}	{2.60492, 6.79881}	{{2.16064, 2.14885}, {3., 12.9628}}	{101.866, 53.908}	inf	4.	201.037	{61.2378, 346.881}	4925.92	1.60404	newly	1.	207.29	{60., 460.}	20629.	2.18373	Reff	1.30442	0.801175	{0.233642, 1.58219}	51.3896	ar	362.	1541.66	{1063., 2115.}	325.873
29	R0	{1.28202, 6.23047}	{2.03459, 4.68145}	{{0.176408, 1.76781}, {3., 7.50542}}	{101.916, 42.9613}	inf	7.	112.548	{11.7592, 352.044}	1513.02	0.988139	newly	13.	125.07	{13., 318.}	871.	0.768572	Reff	1.22561	0.38523	{0.0321212, 0.596245}	69.4927	ar	391.	1652.43	{1428., 2001.}	322.616
30	R0	{1.28177, 8.83252}	{2.24373, 6.82834}	{{0.946562, 2.33772}, {3., 15.816}}	{90.4376, 52.1942}	inf	15.	269.815	{66.57, 516.476}	1698.76	1.15506	newly	15.	346.54	{58., 766.}	2211.2	1.19394	Reff	1.24332	1.05311	{0.392367, 1.79064}	32.6779	ar	427.	1357.12	{715., 1946.}	218.197
31	R0	{1.35886, 5.97708}	{2.52789, 7.00181}	{{1.72607, 2.53035}, {3., 13.106}}	{87.3626, 49.7649}	inf	20.	195.516	{82.6824, 338.463}	880.554	0.881824	newly	20.	193.52	{43., 468.}	871.	0.83528	Reff	1.27733	0.644789	{0.207236, 1.08761}	57.6325	ar	491.	1579.63	{1205., 1949.}	221.717
32	R0	{1.27601, 17.9804}	{2.08882, 4.85541}	{{0.20297, 1.61679}, {3., 7.51973}}	{101.888, 73.0468}	inf	14.	109.58	{11.4314, 292.224}	693.181	0.703054	newly	3.	115.18	{10., 290.}	3741.33	1.29172	Reff	1.24028	0.381357	{0.0402057, 0.574768}	70.1404	ar	445.	1628.99	{1206., 1991.}	266.119
33	R0	{1.3368, 11.3235}	{2.21795, 7.57885}	{{1.21389, 4.46419}, {3., 15.2599}}	{76.8445, 48.5168}	inf	17.	135.985	{19.7128, 268.12}	706.785	0.739561	newly	9.	112.8	{17., 269.}	1158.89	0.893335	Reff	1.27531	0.499151	{0.138928, 1.21268}	64.9426	ar	417.	1595.15	{1188., 2037.}	282.53
34	R0	{1.21021, 11.3635}	{2.04378, 5.1078}	{{0.219273, 1.73762}, {3., 7.33167}}	{109.398, 60.2715}	inf	11.	102.382	{6.4438, 285.78}	843.907	0.821839	newly	11.	106.85	{8., 281.}	884.636	0.810438	Reff	1.12671	0.366702	{0.0317748, 0.573258}	69.0469	ar	327.	1624.63	{1320., 1919.}	396.829
35	R0	{1.53382, 10.8866}	{2.55784, 7.25747}	{{1.63036, 2.52475}, {3., 12.7411}}	{66.7628, 46.1125}	inf	11.	191.969	{65.5629, 324.905}	1649.08	1.11263	newly	11.	183.26	{45., 360.}	1571.45	1.07245	Reff	1.49547	0.673571	{0.287605, 0.996762}	61.3174	ar	476.	1570.1	{1188., 1970.}	229.853
36	R0	{1.20423, 6.83844}	{2.28963, 7.2208}	{{0.95006, 2.31891}, {3., 15.8657}}	{101.866, 57.1368}	inf	6.	266.955	{64.5249, 528.41}	4349.24	1.54022	newly	7.	331.5	{52., 779.}	4635.71	1.48353	Reff	1.18255	1.07887	{0.388869, 2.02394}	33.4983	ar	416.	1384.03	{768., 1941.}	233.007
37	R0	{1.43629, 10.1548}	{2.07813, 5.04788}	{{0.208609, 1.77663}, {3., 7.33241}}	{86.2663, 57.1189}	inf	10.	111.937	{12.1221, 304.24}	1026.03	0.85128	newly	5.	116.83	{11., 310.}	2239.8	1.1006	Reff	1.34006	0.376705	{0.0305672, 0.597592}	72.6036	ar	367.	1646.83	{1385., 1923.}	348.728
38	R0	{1.37504, 8.93428}	{2.25372, 7.13143}	{{0.949631, 2.3425}, {3., 15.8265}}	{82.6224, 51.4583}	inf	15.	263.308	{67.024, 519.322}	1655.39	1.1389	newly	14.	329.1	{57., 799.}	2252.14	1.18802	Reff	1.32829	1.04833	{0.372635, 1.82792}	36.5692	ar	483.	1372.52	{762., 1937.}	184.774
39	R0	{1.51637, 9.36742}	{2.5697, 8.29085}	{{1.72015, 3.05175}, {3., 14.5155}}	{69.4639, 44.7944}	inf	28.	182.75	{64.7611, 331.486}	559.27	0.711488	newly	20.	154.02	{31., 285.}	675.4	0.735335	Reff	1.37686	0.634404	{0.205851, 1.0123}	62.2787	ar	441.	1560.98	{1086., 2091.}	253.964
40	R0	{1.23658, 9.82869}	{2.32666, 9.76108}	{{1.29638, 2.2974}, {3., 15.8921}}	{95.6491, 38.7397}	inf	23.	248.754	{66.6959, 421.197}	987.321	0.945628	newly	14.	244.83	{53., 587.}	1655.5	1.08767	Reff	1.18712	1.32131	{0.235752, 2.23044}	48.3773	ar	414.	1331.09	{725., 1845.}	221.519
41	R0	{1.24247, 5.4242}	{2.30104, 9.53967}	{{0.810792, 2.14153}, {3., 14.2397}}	{98.7766, 96.4766}	inf	9.	192.677	{43.3153, 413.782}	2042.44	1.19346	newly	11.	221.86	{23., 632.}	1920.36	1.07957	Reff	1.22135	1.4406	{0.209388, 2.7581}	57.255	ar	345.	1137.16	{552., 2039.}	230.841
42	R0	{1.28208, 4.40223}	{2.31377, 9.28065}	{{1.528, 2.21466}, {3., 15.5387}}	{86.4723, 122.702}	inf	3.	240.077	{70.4878, 375.065}	7903.77	1.775	newly	6.	250.3	{46., 635.}	4074.	1.41956	Reff	1.26028	1.27814	{0.235979, 2.23197}	45.733	ar	339.	1341.33	{685., 1856.}	295.673
43	R0	{1.56773, 6.50474}	{1.77628, 9.14321}	{{0.376531, 2.00079}, {3., 18.2706}}	{60.1783, 88.3198}	inf	6.	77.4804	{15.1906, 152.283}	1198.63	0.967298	newly	5.	173.65	{10., 539.}	3381.8	1.18355	Reff	1.55205	1.4222	{0.327997, 2.68006}	51.4484	ar	498.	647.02	{142., 992.}	69.2209
44	R0	{1.10489, 6.01941}	{2.57138, 5.70117}	{{2.14471, 0.622172}, {3., 9.70558}}	{134.026, 40.8364}	inf	18.	55.8833	{11.7512, 109.927}	225.443	0.428849	newly	22.	100.33	{10., 196.}	379.591	0.425277	Reff	1.03418	0.576639	{0.115341, 1.3456}	65.8518	ar	346.	1839.54	{1650., 2371.}	431.659
45	R0	{1.25888, 14.1926}	{2.2199, 5.25002}	{{0.62117, 1.45452}, {3., 12.5357}}	{103.546, 63.8268}	inf	42.	51.2163	{4.73104, 55.1688}	99.1296	0.42577	newly	17.	58.82	{4., 64.}	278.588	0.447332	Reff	1.09523	0.405333	{0.0985728, 0.636852}	68.3644	ar	471.	1778.88	{1637., 1875.}	277.682
46	R0	{1.45292, 16.616}	{2.23594, 6.84527}	{{0.972468, 2.32019}, {3., 15.8423}}	{72.8934, 61.5004}	inf	9.	265.747	{64.6133, 500.583}	2852.75	1.36608	newly	4.	333.	{53., 768.}	8225.	1.74053	Reff	1.43258	1.01405	{0.362516, 1.82062}	40.6601	ar	494.	1321.46	{686., 1780.}	168.324
47	R0	{1.22528, 16.6033}	{2.53164, 7.4059}	{{1.62919, 2.5268}, {3., 12.7953}}	{106.618, 56.997}	inf	12.	195.226	{60.5976, 339.437}	1533.48	1.09022	newly	3.	175.61	{33., 358.}	5757.67	1.58556	Reff	1.19955	0.680494	{0.264319, 1.03213}	55.8699	ar	368.	1582.79	{1202., 2133.}	330.106
48	R0	{1.1669, 6.92715}	{2.29779, 5.14987}	{{0.672128, 1.01324}, {3., 13.1886}}	{117.432, 62.2937}	inf	9.	64.6279	{5.57329, 152.357}	633.205	0.618224	newly	10.	81.74	{6., 111.}	733.6	0.590289	Reff	1.05138	0.431407	{0.109431, 0.667379}	64.3531	ar	416.	1811.68	{1645., 2011.}	335.5
49	R0	{1.32749, 16.0887}	{2.24538, 7.49268}	{{1.19029, 4.32839}, {3., 16.8088}}	{78.7514, 56.7153}	inf	11.	135.338	{37.8561, 274.557}	1131.33	0.954209	newly	7.	114.07	{26., 212.}	1530.14	1.0258	Reff	1.29165	0.456172	{0.130317, 1.18592}	67.107	ar	353.	1589.26	{1159., 2052.}	350.215
50	R0	{1.29636, 15.2609}	{2.44004, 7.27131}	{{1.60623, 4.01407}, {3., 14.1808}}	{91.6987, 52.3795}	inf	39.	49.7309	{4.64669, 79.8925}	103.535	0.393395	newly	16.	51.12	{3., 94.}	265.125	0.433006	Reff	1.16283	0.498422	{0.0739808, 0.69375}	64.4444	ar	470.	1818.21	{1632., 2210.}	286.853
